# Chromosomal analysis of blastocyst derived from monopronucleated ICSI zygotes: approach by double trophectoderm biopsy

**DOI:** 10.5935/1518-0557.20170039

**Published:** 2017

**Authors:** Silvia Mateo, Francesca Vidal, Lluc Coll, Anna Veiga, Montserrat Boada

**Affiliations:** 1 Reproductive Medicine Service, Department of Obstetrics, Gynaecology and Reproduction, Women's Health Dexeus, Barcelona, Spain.; 2 Cell Biology Unit, Faculty of Biosciences, Universitat Autònoma de Barcelona, Barcelona, Spain.; 3 Stem Cell Bank, Centre for Regenerative Medicine, Barcelona, Spain.

**Keywords:** monopronucleated zygote, trophectoderm biopsy, aCGH, FISH

## Abstract

**Objective:**

This study aims to increase the knowledge about monopronucleated ICSI-derived blastocysts, analyzing trophectoderm biopsies by aCGH and FISH to evaluate their chromosome constitution.

**Methods:**

Fifteen monopronucleated ICSI-derived blastocysts were studied. Double trophectoderm biopsy was performed and analyzed by FISH and aCGH. The blastocysts were classified according to chromosome constitution. Disagreements between the two techniques were assessed.

**Results:**

Results obtained after FISH and aCGH analyses showed the following: 20% (3/15) and 60% (9/15) diploid females, respectively; 26.7% (4/15) and 26.7% (4/15) diploid males, respectively; and 53.3% (8/15) and 13.3% (2/15) mosaics, respectively. No mosaic male embryos were found using FISH or aCGH. There were disagreements in 40% (6/15) of the cases due to the higher detection of mosaicism by FISH compared to aCGH.

**Conclusions:**

The combination of FISH and aCGH has been shown to be a suitable approach to increase the knowledge about monopronucleated ICSI-derived embryos. FISH analysis of blastocysts derived from monopronucleated ICSI zygotes enabled us to conclude that aCGH underestimates haploidy. Some diploid embryos diagnosed by aCGH are in fact mosaic. In cases where these embryos would be used for reproductive purposes, extra analysis of parental genome origin is recommended.

## INTRODUCTION

The finding of a unique pronucleus is evidence that some error has occurred in the fertilization process. The questions are: why does this happen, how do the resulting embryos appear, and can they be considered for reproductive purposes? Many authors contributed to this research to expand the knowledge about monopronucleated zygotes (1PN) (Munné *et al.*, 1993; Staessen *et al.*, 1993; Levron *et al.*, 1995; Sultan *et al.*, 1995; Staessen & Van Steirteghem, 1997; Otsu *et al.*, 2004; Van Der Heijden *et al.*, 2009; Mateo *et al.*, 2013; 2017; Azevedo *et al.*, 2014; Rosenbusch, 2014).

The genetic composition of monopronucleated ICSI zygotes may have different parental origin, and the mechanisms leading their formation can be diverse:

i) Gynogenetic embryos derived from 1PN ICSI zygotes could be the result of a parthenogenetic activation, without the participation of the paternal genome. This could be due to the extrusion of spermatozoa to the perivitelline space, the absence of decondensation of the paternal nucleus, or premature paternal chromosome condensation (Flaherty *et al.*, 1998). If only one polar body (PB) is present, the embryo would be diploid. When two PB are found, the embryo will be haploid, or diploid if endoreduplication has occurred.

ii) Monopronucleated zygotes can also originate androgenetic embryos when there is correct formation of the male pronucleus, avoiding the formation of the female pronucleus. This could be due to the complete extrusion of maternal genome in the second polar body, or due to the maintenance of the meiotic spindle of the oocyte (Azevedo *et al.*, 2014; Kai *et al.*, 2015).

iii) Monopronucleated zygotes with biparental origin could arise from the formation of a unique pronucleus, including maternal and paternal genomes (Levron *et al.*, 1995; Van Der Heijden *et al.*, 2009; Kai *et al.*, 2015). In this case, the union of the maternal and paternal genetic materials could be produced prior to the membrane formation due to the tight proximity of the spermatozoa and the oocyte spindle, or due to the formation of two pronuclei and a subsequent fusion in one pronucleus (Levron *et al.*, 1995; Flaherty *et al.*, 1998; Meseguer, 2016). The finding of an asynchronous pronuclei has also been reported before, and could be another reason for finding a unique pronucleus (Staessen *et al.*, 1993) when time-lapse methodology is not used.

Concerning the possible reproductive use of these embryos, there are different considerations depending on whether they arise from conventional IVF (cIVF) or intracytoplasmic sperm injection (ICSI). While the former is often accepted for clinical use, embryos from 1PN ICSI zygotes are usually discarded due to the reported high incidence of chromosomal abnormalities.

A recently published paper, reporting that some of these zygotes can reach the blastocyst stage, being euploid and resulting in the birth of a healthy child, suggests that they could be used for reproductive purposes in certain cases (Mateo *et al.*, 2017).

A diagnosis of euploidy must be mandatory to consider any 1PN ICSI-derived embryo for transfer. Currently, the Pre-implantation Genetic Screening (PGS) approach used in most centers is addressed to a Comprehensive Chromosomal Screening (CCS) by array Comparative Genome Hybridization (aCGH) in biopsied trophectoderm cells. However, the main limitation of aCGH is its suitability to ascertain the ploidy status of the studied embryos (Gutiérrez-Mateo *et al.*, 2011; Scriven, 2013), and this is an important limitation when there is risk of having haploid embryos in the cohort studied. Fluorescent *in situ* hybridization (FISH), although being set aside by most groups since the implementation of aCGH, could easily provide information about embryo ploidy.

The objective of this study is to analyze trophectoderm biopsies by aCGH and FISH, to assess to which extend the use of aCGH may lead to an underestimation of haploidy, and to gain knowledge about the chromosome content of embryos coming from 1PN ICSI zygotes.

## MATERIAL AND METHODS

Fifteen blastocysts from monopronucleated (1PN) zygotes with two polar bodies (2PB) obtained after ICSI were analyzed. Patients agreed to donate vitrified blastocysts from 1PN 2PB ICSI zygotes, after being informed that these embryos were considered not suitable for reproduction, because of their reported high incidence of chromosomal abnormalities. Patients signed the corresponding written informed consent. In all cases, embryos from 2PN 2PB zygotes were available for transfer. The study was approved by the institutional review board of the center.

ICSI was performed at 40h post HCG administration. After ICSI, zygotes were cultured in LifeGlobal total^®^ media (LifeGlobal^®^) and were placed in a time-lapse incubator (EmbryoScope^®^-Vitrolife). Images of 5 focal planes were acquired at every 15 minutes. Dynamic monitoring allowed the presence of only a single PN to be confirmed, and either asynchronous 2PN formation or 2PN fusion to be excluded.

Monopronucleated ICSI zygotes were maintained in culture until blastocyst formation. Blastocysts were vitrified and, after being rejected for reproductive purposes, analyzed for research.

### Vitrification and warming

Kitazato media were used for vitrification/warming according to the manufacturer's protocol and cryotop^®^ (Kitazato^®^) was used as support. After warming, all blastocysts were cultured in LifeGlobal total^®^ media for 30 minutes.

### Blastocyst biopsy

For zona drilling, the embryos were moved to LifeGlobal Total^®^ w/HEPES. A laser (NaviLase, OCTAX Microscience GmbH) attached to a microscope (U-LH100L-3, Olympus^®^) was used. Three laser pulses of 1.3ms were applied in the zona pellucida of the blastocyst, opposite to the inner cell mass. After zona drilling, the blastocysts were placed in LifeGlobal total^®^ media (LifeGlobal^®^) for at least 8 hours, to facilitate the trophectoderm (TE) herniation for later biopsy. Biopsy of two different TE fragments of each blastocyst were collected by aspiration (between 4 and 16 cells each, depending on the characteristics of the TE).

### Chromosomal analysis

FISH was performed in one of the TE fragments and aCGH analysis in the other.

### FISH analysis

TE cells were washed in phosphate-buffered saline solution (PBS) (6% human serum albumin) and were fixed on a slide by adding Carnoy's solution (methanol-acetic acid; 3:1). The fixation procedure was performed under an inverted microscope and was adapted to the fragment's characteristics for accurate cell spreading. Biopsies were individually fixed on different slides to avoid cell contamination from other blastocysts. After fixation, the slides were left to dry and they were stored at -4°C until they were processed for FISH analysis.

Since the objective of the FISH analysis was ploidy assessment, the slides were processed for FISH using a commercial probe panel, specific for chromosomes X, Y and 18 (AneuVysion Multicolor DNA Probe Kit, Vysis CEP 18/X/Y, Abbott Molecular) according to the manufacturer's protocols. Interphase nuclei were evaluated using an Olympus BX-61 fluorescent microscope (Olympus^®^) equipped with specific filters for FITC, Cy3, and Aqua and a multiband pass filter (DAPI/FITC/Texas Red). Overlapped nuclei, metaphase figures and chromatin fragments were discarded from the analysis. Embryos were further classified according to their chromosomal constitution. According to FISH results, the embryos were classified into haploid (H), diploid (D) or mosaic (M), and further gender identified as f (female) or m (male) depending on the results of the cells analyzed. According to this, Df and Dm were those embryos with all the cells analyzed containing two chromosomes 18 and XX or XY, respectively. Mf and Mm corresponded to those mosaic embryos with more than one cell line but at least one cell with two copies of chromosome 18 and XX or XY respectively. H were those embryos with all the cells analyzed with one chromosome 18 and one sex chromosome.

### aCGH analysis

TE cells were washed separately in four drops of 10µl of PBS/PVA solution and were transferred to a PCR tube with a drop of 0.1µl of PBS solution. The PCR tubes with the samples were stored at -80°C until they were processed. The trophectoderm cells were processed for DNA amplification and aCGH analysis using 24 sure kit and Fluorescent labelling system (Illumina^®^), according to the manufacturer's protocols. Hybridization results were processed and analyzed with the BlueFuse software (Illumina^®^). The embryos were further classified according to their chromosomal constitution. According to aCGH results, the embryos were classified into diploid (D) or mosaic (M), and further gender identified as f (female) or m (male). According to this classification, Df and Dm were those embryos showing diploid female or diploid male homogeneous chromosomal complements, respectively. Mf and Mm corresponded to mosaic embryos with diploid female or male cell lines, respectively.

## RESULTS

Fifteen 1PN 2PB-derived blastocysts were successfully thawed (100% survival rate) and all had re-expanded their blastocoel cavity after 8h of culture.

Results from FISH and aCGH analysis and embryo classification are detailed in Tables 1 and 2 respectively.

**Table 1:** Results from FISH analysis, embryo classification and percentage of diploid and haploid cells in 1PN 2PB-derived blastocyst. Df: diploid female; Dm: diploid male; Mf: mosaic female; Mm: mosaic male; M: mosaic for the sex chromosomes

EMBRYO CODE	FISH RESULTS [number of cells]	% DIPLOID CELLS	% HAPLOID CELLS	FISH CLASSIFCATION
E1	XX1818 [12]	100% (12/12)	0%	Df
E2	XX1818[6]; X18 [1]	85.7% (6/7)	14.3% (1/7)	Mf
E3	XX1818 [3]; X18 [2]	60% (3/5)	40% (2/5)	Mf
E4	XX1818 [7]; XXXX18181818[1]	87.5% (7/8)	0%	Mf
E5	X18 [4]; XXXX18181818[4]; XX1818[2]	20% (2/10)	40% (4/10)	Mf
E6	XY1818 [5]	100% (5/5)	0%	Dm
E7	X18 [5]; XX1818 [1]; XXXX18181818 [1]	14.3% (1/7)	71.4% (5/7)	Mf
E8	XY1818 [6]	100% (6/6)	0%	Dm
E9	X18 [15]; XX1818 [1]	6.3% (1/16)	93.8% (15/16)	Mf
E10	XY1818 [4]	100% (4/4)	0%	Dm
E11	XY1818 [4]	100% (4/4)	0%	Dm
E12	X18 [7]; XY1818 [5]; XX1818 [2]	50% (7/14)	50% (7/14)	M
E13	XX1818 [4]; X18 [1]	80% (4/5)	20% (1/5)	Mf
E14	XX1818 [5]	100% (5/5)	0%	Df
E15	XX1818 [13]	100% (13/13)	0%	Df

**Table 2:** Results from aCGH analysis and embryo classification of 1PN 2PB-derived blastocysts. Df: diploid female; Dm: diploid male; Mf: mosaic female; Mm: mosaic male; M: mosaic for the sex chromosomes. (*) Non-mosaic aneuploid embryo

EMBRYO CODE	aCGH RESULTS	aCGH CLASSIFICATION
E1	46, XX	Df
E2	46, XX	Df
E3	46, XX	Df
E4	46, XX/45, XX (-8)	Mf
E5	46, XX	Df
E6	47, XY (+22)	Dm*
E7	46, XX	Df
E8	46, XY (-16, +21)	Dm*
E9	46, XX	Df
E10	46, XY	Dm
E11	46, XY	Dm
E12	46, XY/46, XXY (-6)	M
E13	46, XX	Df
E14	46, XX	Df
E15	46, XX	Df

Concerning FISH results, haploid, diploid female, diploid male and tetraploid cells were found. Diploid cells were found in all the embryos studied. Haploid cell lines were observed in 46.7% of the embryos (7/15) and tetraploid cells were found in 20% of the embryos (3/15). No aneuploidy for any of the three chromosomes analyzed was observed (Table 1).

According to FISH results, 20% (3/15) of the embryos were classified as diploid female, 26.7% (4/15) as diploid male and 46.7% (7/15) as mosaic female. One embryo (E12) was classified as mosaic (6.7%; 1/15) with coexisting diploid male, diploid female and haploid cell lines. No haploid embryos and no mosaic diploid male embryos were found (Table 1).

Concerning aCGH, the embryos were classified as diploid female in 60% (9/15), 26.7% (4/15) as diploid male, one mosaic female (6.7%) and one mosaic for the sex chromosomes (6.7%). Aneuploidy was found in 26.7% of the embryos (4/15). In two diploid male embryos (E6 and E8), the results were consistent with homogeneous aneuploidy for all the cells analyzed. The other two aneuploidy embryos (E4 and E12) were mosaic (Table 2).

Total agreement between FISH and aCGH results was found in 60% (9/15) of the embryos, corresponding to embryos diagnosed by FISH as diploid (E1, E6, E8, E10, E11, E14, E15) and mosaic embryos E4 and E12, the latter with mosaicism for the sex chromosomes. Similar percentages between male and female embryos were observed in embryos diagnosed as diploid, representing 46.7% of the total embryos (Table 3).

**Table 3:** Agreement between classifications obtained after FISH and aCGH analysis of 1PN 2PB-derived blastocysts. H: Haploid; Df: diploid female; Dm: diploid male; Mf: mosaic female; Mm: mosaic male; M: mosaic for the sex chromosomes

aCGH								
		H	Df	Dm	Mf	Mm	M	TOTAL
FISH	H	0	0	0	0	0	0	0
	Df	0	3	0	0	0	0	3
	Dm	0	0	4	0	0	0	4
	Mf	0	6	0	1	0	0	7
	Mm	0	0	0	0	0	0	0
	M	0	0	0	0	0	1	1
	TOTAL	0	9	4	1	0	1	60% (9/15)

There was no agreement in 40% of the embryos (6/15). Seven mosaic female embryos were detected by FISH and, six of them, were classified as diploid females by aCGH. Classification disagreements between the two techniques were due to a higher detection of mosaicism by FISH when compared to aCGH (53.3%; 8/15 vs. 13.3%; 2/15). The remaining embryo was classified as mosaic female, an agreement between both techniques (Table 3).

In all the embryos analyzed, gender assignment was concordant between the two techniques. Special attention should be paid to embryo E12, which showed mosaicism for the sex chromosomes (Table 3).

## DISCUSSION

There are few papers where monopronucleated ICSI zygotes were cultured until blastocyst stage (Otsu *et al.*, 2004; Mateo *et al.*, 2013; 2017; Yao *et al.*, 2016) and all came from a small study cohort. The reason for the low number of blastocysts included in the present study was the low incidence of monopronucleated ICSI zygotes in the IVF program (3.1%, unpublished data, Dexeus Women's Health), the restrictive 1PN 2PB assessment using time-lapse and the low development potential of 1PN 2PB ICSI zygotes with regard to reaching the blastocyst stage (14.8%, Mateo et al., 2013; 3.6%, Yao et al., 2016; 3.4%, Mateo et al., 2017).

The disagreements found between the techniques used can be explained because a high number of mosaic female embryos detected by FISH were misclassified by aCGH, due to their failure in detecting haploid and tetraploid cells. Even so, it cannot be ruled out that the disagreements were the result of analyzing two different TE fragments; it seems unlikely because the most commonly observed mosaicism involved ploidy alterations. The coexistence of diploid, haploid and tetraploid cells has been already reported in embryos from monopronucleated zygotes as well as in those which were normally fertilized (Staessen & Van Steirteghem, 1997; Daphnis *et al.*, 2005). Female embryos showing a high percentage of diploid cells, and few haploid and tetraploid cells could originate from fertilization involving paternal and maternal genomes. The presence of tetraploid cells is a common phenomenon and attributed to the endoreduplication of diploid cells (Daphnis *et al.*, 2005). The presence of few haploid cells could be explained by the formation of binucleate cells with a subsequent cytokinesis (Delhanty *et al.*, 1997). Mosaic female embryos with a high percentage of haploid cells could have originated from oocyte parthenogenetic activation. The embryo initially would be haploid, and later some cells will turn into diploid cells by endoreduplication.

With regards to these findings, embryos in which all the cells analyzed were diploid, those mosaics with diploid/tetraploid cells and diploid/tetraploid/haploid cells with a low presence of haploid cells (<50%) could be considered fertilized; this accounts for 86.7% (13/15). The percentage of diploidy has been shown to be lower when chromosomal assessment is performed in early development (Sultan *et al.*, 1995; Staessen & Van Steirteghem, 1997; Van Der Heijden *et al.*, 2009; Mateo *et al.*, 2013) but higher, when it is performed in blastocysts (Mateo *et al.*, 2013). The selection against haploid embryos through culturing up to the blastocyst stage could explain these differences (Gras & Trounson, 1999).

Embryo E12 needs special consideration as coexistent haploid, diploid female and diploid male cells have been observed. These cells could come from the rescue of a triploid XXY embryo, resulting in one diploid male cell line and another haploid cell line, with subsequent diploidization of some haploid cells. Although the rescue of a triploid embryo has been reported after dispermic fertilization, it has not been confirmed after ICSI (Golubovsky, 2003). The origin of this triploid XXY embryo could be the result of either the fecundation of the oocyte by a diploid spermatozoa or by a spermatozoa carrying the Y chromosome together with endoreduplication of the oocyte genome (Rosenbusch, 2008; 2014).

The combination of FISH and aCGH techniques has been a suitable approach to expand the knowledge 1PN 2PB ICSI-derived embryos, and to allow the detection of mosaicism, which would not be detected by applying only one technique. It has been shown that the application of aCGH for analysis may lead to an underestimation of embryos with a high proportion of haploid cells and fully haploid embryos that could compromise the correct development of further pre-implantation stages. Furthermore, it has been demonstrated that FISH approach is not a safe option for the analysis of 1PN 2PB-derived embryos for clinical use, as it cannot distinguish between fertilized and non-fertilized diploidized embryos. Even more, FISH analysis where only few chromosomes are analyzed can underestimate the impact of aneuploidy. This is of special importance in 1PN-derived embryos, as high aneuploidy rates have been observed among them (Mateo *et al.*, 2013). Consequently, we recommend an initial analysis with a CCS technique to evaluate the euploidy of 1PN-derived embryos.

In case those embryos would be needed for reproductive purposes, blastocysts diagnosed as diploid males could be considered, as the presence of the Y chromosome ensures the paternal genome contribution. Despite that, the possibility of an androgenetic origin, with the contribution of only the paternal genome, is not ruled out, although it may seem like a very rare phenomenon (Azevedo *et al.*, 2014; Kai *et al.*, 2015). As aCGH was concordant with FISH in gender assignment, the use of FISH for detecting male embryos will not be necessary. Considering the possible use of diploid female embryos or female mosaics with a high percentage of diploid cells, the use of both FISH and aCGH would not solve the dilemma, as the possible origin by parthenogenesis followed by endoreduplication is not ruled out. The low frequency of uniparental disomy observed in human blastocysts, which is equivalent to those observed in live births, indicates that the presence of uniparental disomy is a rare phenomenon, but it could increase among abnormally fertilized embryos (Gueye *et al.*, 2014; Xu *et al.*, 2015). For better assessment, despite an euploid diagnosis after aCGH has been achieved, other diagnostic methods covering the analysis of parental genome origin should be performed in blastocysts derived from 1PN 2PB ICSI zygotes.

## CONCLUSIONS

The additional use of FISH in the analysis of blastocysts derived from 1PN 2PB ICSI zygotes enabled the conclusion that aCGH underestimates haploidy. Furthermore, some diploid embryos diagnosed by aCGH are, in fact, mosaic. In cases where 1PN 2PB ICSI-derived embryos would be used for reproductive purposes, despite an euploid diagnosis after aCGH has been achieved, an extra analysis of parental genome origin should be performed.
